# Ripening-Related Changes in Color and Bioactive Compounds of *Diospyros kaki*: Preliminary Insights on Its Antifungal Activity

**DOI:** 10.3390/foods14081332

**Published:** 2025-04-11

**Authors:** Francesco Cairone, Letizia Angiolella, Francesca Bertini, Antonia Iazzetti, Giancarlo Fabrizi, Stefania Petralito, Stefania Cesa, Giovanna Simonetti

**Affiliations:** 1 Department Chemistry and Technologies of Drug, University “La Sapienza” of Rome, P.le Aldo Moro 5, 00185 Rome, Italy; francesco.cairone@uniroma1.it (F.C.); francesca.bertini@uniroma1.it (F.B.); giancarlo.fabrizi@uniroma1.it (G.F.); stefania.petralito@uniroma1.it (S.P.); 2 Department of Public Health and Infectious Diseases, University “La Sapienza” of Rome, P.le Aldo Moro 5, 00185 Rome, Italy; letizia.angiolella@uniroma1.it; 3 Department of Basic Biotechnological Sciences, Intensivological and Perioperative Clinics, Catholic University of the Sacred Heart, L.go F. Vito 1, 00168 Rome, Italy; antonia.iazzetti@unicatt.it; 4 Department of Environmental Biology, University “La Sapienza” of Rome, P.le Aldo Moro 5, 00185 Rome, Italy; giovanna.simonetti@uniroma1.it

**Keywords:** *Diospyros kaki*, carotenoids, polyphenols, CIEL*a*b* analysis, HPLC-DAD analysis, anti-*Candida* activity, *Candida* biofilm

## Abstract

*Diospyros kaki* L. is acknowledged for its extraordinary phytotherapeutic properties due to the presence of polyphenols, carotenoids, and flavonoids such as β-cryptoxanthin and rutin. These compounds are largely distributed in the skin and flesh of the fruit. In this study, the different parts of persimmons were analyzed (whole fruit, peels, and flesh), aiming at determining total carotenoid and flavonoid content through selective extraction and HPLC-DAD analysis. Obtained by a one-pot double phase extraction, organic and aqueous extracts were submitted to colorimetric analyses and tested for their antifungal activity. Results indicated that carotenoid and flavonoid content varied with sample maturity, and colorimetry proved to be an effective predictor of pigments’ composition. The strongest antifungal and antibiofilm activity has been demonstrated for the hydroalcoholic extracts of the unripe whole fruit and flesh. Preliminary results suggest their potential application in preventing *Candida* infections by inhibiting their establishment. Although further studies are needed, these results open the way to the possible use of the extracts as additives in foods or in the preparation of pharmaceutical formulations for the prevention of infections caused by *Candida albicans*, helping to reduce the use of synthetic biocidal products.

## 1. Introduction

*Diospyros kaki* L., commonly known as khaki, is a fruit native to East Asia, known for its high content of bioactive compounds. This plant is popular for its distinctive aroma and the nutritional benefits associated with the consumption of the fruit [1]. Being a member of the *Ebenaceae* family, this plant is characterized by large leaves and single or small, grouped flowers, which produce bright orange or red fruits. These fruits, particularly known for their high sugar content and sweet taste, have high nutritional and biochemical value [2]. The peels, which account for approximately 10–15% of the fresh fruit’s weight, are rich in bioactive compounds, particularly polyphenols and carotenoids. Notably, polyphenols—primarily represented by rutin—constitute up to 30% of the total phenolic content in the whole fruit. Among the carotenoids, β-cryptoxanthin, lutein, and β-carotene are the most abundant, with β-cryptoxanthin possibly accounting for up to 40% of the total carotenoids [2,3]. These phytochemical components valorize kaki as a functional food but also suggest innovative applications for the utilization of its waste with a view to sustainability and by-product utilization.

Several studies [3,4,5] have shown that regular consumption of khaki helps to reduce LDL cholesterol levels and improve endothelial function, therefore, reducing the risk of atherosclerosis and cardiovascular disease. In addition, carotenoids, particularly β-cryptoxanthin and lutein, have been associated with visual health benefits. Rutin and other flavonols, found in the fruit’s peel and flesh, also show potential anti-carcinogenic effects [5,6].

Among its antimicrobial properties, aqueous extracts of *Diospyros kaki* exhibit antibacterial and antidiarrheal effects, as methanolic extracts of different kaki species have been shown to inhibit human bacterial and fungal pathogens [7,8]. In recent years, there has been growing interest in natural sources of bioactive compounds with antimicrobial potential, particularly to counter the rise of fungal strains resistant to conventional treatments. Previous studies have demonstrated the antimicrobial activity of *Diospyros kaki* extracts against both Gram-positive and Gram-negative bacteria, which act by inhibiting biofilm formation, disrupting energy metabolism in pathogens, and modulating host inflammatory responses [9]. In addition, recent studies have also demonstrated that natural food extracts, such as *Diospyros kaki*, rich in polyphenols including flavanols, flavonols, tannins, and others, exhibit promising antifungal activity, particularly against *Candida albicans* [7,10]. In particular, the condensed tannins present in the fruit are thought to act by destabilizing the fungal cell membrane and inhibiting biofilm formation [11].

Gut microbiota imbalances can lead to diarrhea and other dysbiosis-related diseases, often associated with opportunistic pathogens such as *Candida albicans*. In immunocompromised individuals, *Candida* infections pose a therapeutic challenge due to the fungus’s ability to develop resistance to conventional antifungal drugs. This risk is particularly high in patients with inflammatory bowel disease (IBD), where *Candida* species, capable of transitioning between yeast and hyphal forms, are the most common fungal pathogens in the gastrointestinal tract [8,12]. Moreover, emerging evidence suggests that *Candida* infections can be acquired from the hospital environment, with a relatively high incidence of carriage on the skin of healthcare workers. Their hands are considered a key factor in *Candida* colonization and transmission, highlighting the importance of effective infection control measures to limit the spread of this pathogen [12]. A key virulence factor of *C. albicans* is its ability to form biofilms, whose formation represents a form of resistant survival in the environment [13,14]. Cells that adhere to surfaces and become embedded in the matrix produced by microorganisms are significantly more resistant to antifungal treatments. Moreover, the biofilm serves as a reservoir of cells that can disperse and contribute to the spread of infection. Preventing biofilm formation on both biotic surfaces, such as the intestine or skin, is a strategy to prevent the establishment of infection [15,16]. Given the promising bioactive properties of persimmon (*Diospyros kaki*), we hypothesized that its extracts may exhibit antifungal activity against *Candida albicans*, inhibiting both planktonic growth and biofilm formation. To investigate these properties, we characterized the polyphenolic and carotenoid content of persimmons at different ripening stages and in various fruit parts (whole fruit, peel, and flesh). By analyzing how these bioactive compounds are distributed, we seek to better understand their potential health benefits and explore their application in the development of formulations for the prevention of *Candida* infections. The antifungal efficacy of the obtained persimmon extracts against *C. albicans* planktonic cells and biofilms is contextualized in a larger and growing concern in healthcare settings [17,18]. Identifying the most bioactive extracts and their optimal maturation stage could support the development of plant-based antifungal solutions, reducing dependence on synthetic biocides. Furthermore, to validate these findings beyond in vitro studies, the *Galleria mellonella* infection model was used to assess the extracts’ effectiveness. Lastly, the correlation between color and carotenoid content suggests that colorimetric analysis could serve as a useful tool for assessing phytocomplex quality, in the broader perspective of valorizing *Diospyros kaki* [19].

## 2. Materials and Methods

### 2.1. Chemicals

The solvents used for the analytical procedures (ethanol 96%, *n*-hexane, acetic acid, acetone, methanol, ethyl acetate, and double-distilled water) and the analytical standards (lutein and rutin) were purchased from Merck Life Science s.r.l., Milan, Italy. The persimmon fruits used for the protocol, classified as VFNA (non-astringent fertilization variable, the most diffuse Italian cultivar, for its non-astringent characters), were purchased from a local supermarket in Rome and were granted a label indicating they come from organic farming present in the Lazio region (Velletri, Rome, Italy).

### 2.2. Sample Preparation

**Figure 1:**
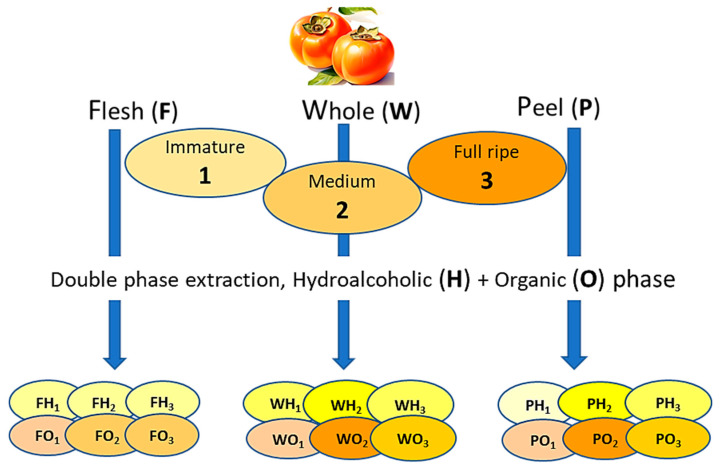
Schematic representation of the double-phase extraction process applied to persimmon samples, flesh (F), whole fruit (W), and peel (P) at different ripening stages. The fruit samples were categorized into three maturation stages: Immature (1), Medium (2), and Full Ripe (3). The obtained hydroalcoholic (H) and organic extracts (O) were subjected to different analytical procedures.

The persimmon fruits were categorized into immature (1), medium ripening (2), and mature (3), based on their degree of ripeness and harvest, and stored at 4 °C until analyses were performed. The fruits were washed, dried, and peeled. Three samples from each fruit were prepared from the whole fruit (W), separated peels (P), and flesh (F), respectively. Then the samples were subjected to homogenization using a domestic blender (Girmi^®^ s.p.a., Brescia, Italy), and the resulting homogenates were immediately subjected to subsequent analysis. An illustrative scheme of the adopted work-up is shown in Figure 1.

### 2.3. Double-Phase Extraction

The homogenized samples were subjected to an optimized double-phase extraction developed in our laboratories [20]. Approximately 20 g of sample was extracted using a 1:1 (*v*/*v* ratio) mixture of n-hexane and a hydroalcoholic solution (ethanol and distilled water acidified with 5% acetic acid, in a 70/30 *v*/*v* ratio). The extraction was performed at a 1:2 *w*/*v* ratio with the sample for 3 h at room temperature, protected from light and under magnetic stirring. The two phases, a hydroalcoholic fraction (H) and an organic fraction (O), were separated and concentrated under vacuum and stored at 4 °C until further analyses were performed.

### 2.4. Colorimetric CIEL*a*b* Analysis

The homogenized and extracted samples were submitted to colorimetric CIEL*a*b* analysis with a colorimeter X-Rite MetaVue^TM®^ (X-Rite, Incorporated, Grand Rapids, MI, USA) equipped with a full-spectrum LED illuminant and an observer angle of 45°/0° imaging spectrophotometer. The reflectometer provides relative reflectance values in the 400–700 nm wavelength range and CIEL*a*b* parameters, according to Fraschetti et al. (2023) [21]. CIEL*a*b* parameters are allowed to characterize the chromatic properties of the different samples [22].

### 2.5. HPLC-DAD Analysis

Chromatographic analysis of the obtained extracts was performed with a Perkin-Elmer instrument (Waltham, MA, USA) equipped with a 200-series LC pump, a 200-series photodiode detector (LC200D), and a 200-series autosampler.

The organic extracts were diluted in ethyl acetate (1:5 *w*/*v*) and filtered with a Millex^®^—LG filter (Low Protein Binding Hydrophilic PTFE 0.45 µM Membrane) (Merck Science Life s.r.l, Milan, Italy) before analysis. The protocol follows that reported by Patsilinakos et al. (2018) with a few modifications [19]. An RP18 reversed-phase column (Luna^®^ 3 µm C18-100 Å, 150 × 4.60 nm) and a mobile phase consisting of methanol (A) and acetone (B) in gradient were used. The injection volume was 10 μL, and the flow rate was set at 1 mL/min. The applied method was as follows: 90% (A)—10% (B) to 45% (A)—55% (B) in 15′; 45% (A)—55% (B) to 15% (A)—85% (B) in 20′; 15% (A)—85% (B) to 10% (A)—90% (B) in 5′. Chromatograms were recorded at 450 nm. To quantify the carotenoids in the extracts, a calibration curve of the lutein standard expressed in mg/mL was constructed (y = 13,292x + 2.4625; R^2^ = 0.9999, in the range between 0.04 and 0.8 mg/mL, at an LOD of 0.003 mg/mL and LOQ di 0.01 mg/mL, by analyzing standard solutions at different concentrations, yielding %RSD values below 5%, indicating high method reproducibility).

The obtained hydroalcoholic extracts were diluted in methanol (1:1 *w*/*v*) and filtered with a Millex^®^—LG filter (Low Protein Binding Hydrophilic PTFE 0.45 µM Membrane) (Merck Science Life s.r.l., Milan, Italy) before analysis. The protocol follows that reported in Cairone et al. (2022) with a few modifications [20]. An RP18 reversed-phase column (Luna^®^ 3 µm C18(2) 100 Å; LC Column 150 × 4.60 nm) and a mobile phase consisting of acetonitrile (A) and 5% formic acid water (B) in gradient were used. The injection volume was 10 μL, and the flow rate was set at 1 mL/min. The method that was used was as follows: from 100% B to 85% in 15′, to 55% in 30′. Chromatograms were recorded at 360 nm. To quantify the flavonols present in the extracts, the calibration line of the rutin standard expressed in mg/mL was constructed (y = 13,598x + 33.16; R^2^ = 0.9994, in the range between 0.002 and 0.184 mg/mL, at an LOD of 0.028 mg/mL and LOQ di 0.086 mg/mL, by analyzing standard solutions at different concentrations, yielding %RSD values below 5%, indicating high method reproducibility).

### 2.6. In Vitro Antifungal Evaluation

#### 2.6.1. Antifungal Susceptibility Test

The antifungal activity of the extracts was assessed using the broth microdilution method, following standardized protocols for yeast susceptibility testing (CLSI, 2017) [23] previously employed in other scientific studies [20,24].

*C. albicans* strains ATCC 10231 and ATCC 10261, used in this study, were obtained from the American Type Culture Collection (ATCC, Rockville, MD, USA). Cultures of *C. albicans* were initially grown on Sabouraud Dextrose Agar (Sigma Aldrich, St. Louis, MO, USA) at 35 °C for 24 h to achieve optimal growth. The final inoculum concentration was standardized to 2.5 × 10^3^ cells/mL using a cell counting device.

The hydroalcoholic and organic extracts were initially dissolved in dimethyl sulfoxide (DMSO) and subsequently diluted at least 100-fold in RPMI-1640 medium buffered with MOPS (4-morpholinepropanesulfonic acid) to ensure a stable pH. Tested concentrations ranged from 500 μg/mL to 0.98 μg/mL, prepared through serial dilutions in 96-well microplates. Fluconazole (Merck Science Life s.r.l., Milan, Italy) was tested as a control. Minimal inhibitory concentrations (MICs) were determined by comparing yeast growth in treated wells to untreated control wells after 24 h of incubation. Each experiment was performed in duplicate and repeated at least three times to ensure reproducibility.

#### 2.6.2. Anti-Adhesion Assay

The anti-adhesion properties of the extracts were assessed following previously described methods [25]. Pre-sterilized, flat-bottomed 48-well polystyrene microtiter plates were used to evaluate the inhibition of *Candida* adhesion. A 200 μL suspension of *C. albicans* (1.0 × 10^6^ cells/mL) was added to each well along with extract solutions at concentrations ranging from 500 μg/mL to 31.25 μg/mL. The plates were incubated at 37 °C for 90 min. Following incubation, non-adherent cells were removed by aspirating the suspension and washing the wells twice with PBS. Adherent cells were quantified using the crystal violet (CV) assay [25]. Absorbance was recorded at 590 nm using a microplate reader. Each experiment was performed in triplicate and repeated at least three times to ensure reproducibility.

#### 2.6.3. Antibiofilm Activity

The biofilm formation assay was conducted in flat-bottomed, 48-well microtiter plates as described in previous protocols, with minor modifications [25]. A suspension of *C. albicans* (1.0 × 10^5^ cells/mL) was prepared in RPMI-1640 medium buffered with MOPS and distributed into each well along with 200 μL of the extract solutions at concentrations ranging from 500 μg/mL to 31.25 μg/mL. The plates were incubated at 37 °C for 48 h to allow biofilm development. After incubation, the medium was removed, and non-adherent cells were washed away with PBS. The biomass of the biofilm was quantified using the CV assay. Each experiment was performed in triplicate and repeated at least three times to ensure reproducibility.

### 2.7. In Vivo Antifungal Evaluation

#### *Galleria mellonella* Survival Assay

The survival assay was conducted as outlined by Cairone et al. (2022) [20]. *Galleria mellonella* larvae, each weighing 0.3 ± 0.03 g, were obtained from Bigserpens (Paliano, FR, Italy). This facility maintains its own wax moth colony without adding antimicrobial compounds or hormones to the artificial diet. Groups of 10 larvae were injected into the last left proleg with 10 μL of a *C. albicans* suspension, followed by the administration of extract solutions at concentrations of 500 μg/mL, 250 μg/mL, and 125 μg/mL. Control groups included untreated larvae, larvae pierced and injected with sterile saline, and larvae inoculated with *C. albicans* alone. The larvae were maintained at 37 °C and monitored for over 120 h. The larvae were considered dead when they showed no response to gentle probing with forceps. Each assay was repeated three times, and survival percentages were recorded.

### 2.8. Statistical Analysis

The data obtained were analyzed using the Shapiro–Wilk test to confirm normality and subsequently analyzed with a one-sample *t*-test. Significance values are indicated as follows: *p* < 0.0001 very highly significant (****), 0.0001 ≤ *p* < 0.001 highly significant (***), 0.001 ≤ *p* < 0.01 moderately significant (**). *G. mellonella* survival was displayed via Kaplan–Meier curves with a curve comparison test; *p*-value: *p* < 0.0001 very highly significant (****), 0.0001 ≤ *p* < 0.001 highly significant (***). Statistical data analysis was performed using GraphPad Prism 8 software (GraphPad Software Inc., La Jolla, CA, USA).

## 3. Results and Discussion

Extracts of khaki are rich in carotenoids and flavonols, natural chemicals that play a significant role in antioxidant protection and prevention of various diseases [5]. Polyphenols represent the most abundant class of antioxidants found in human diets, with properties that include anti-inflammatory, cardiovascular, anticancer, and immune modulation activities [26]. Similarly, carotenoids are known for their antioxidant properties and ability to protect vision and promote cardiovascular health. These compounds not only contribute to human health but also play a role in the stability and visual quality of foods, as their concentration is often correlated with fruit coloration, an important attribute for consumer perception [22]. This research is part of a sustainability vision and innovation in agribusiness, contributing to the development of circular economy strategies, capable of reducing waste and adding value to traditional food products.

### 3.1. Extraction Procedures

Extraction yields of hydroalcoholic and organic fractions from whole fruit (W), peel (P), and flesh (F) at various ripening stages show notable differences, highlighting the potential to isolate bioactive compounds [27]. The hydroalcoholic fractions generally yielded higher values when flesh of immature fruits was extracted (FH_1_), achieving the lowest yield of 3.5%; analogously, the mature peels (PH_3_) showed the maximum yield of 13.8% (Table 1). These findings align with previous results, showing that fruit peels, especially in advanced ripening stages, are richer in polyphenolic compounds [28]. The peel’s function as a protective barrier enables it to accumulate significant concentrations of polyphenols and carotenoids, countering oxidative and environmental stressors. The use of ethanol rather than methanol in the hydroalcoholic mixture conferred several advantages, including selectivity, eco-sustainability, and reduced toxicity [29].

The organic fractions demonstrated comparatively lower yields (Table 1), with the highest values observed in medium-ripe peel and whole fruit samples (PO_2_ and WO_2_) at about 0.1%, while the immature flesh (FO_1_) recorded a yield of just 0.01%. Hexane was chosen for its efficiency in isolating carotenoids, according to [16], which exploited the selectivity of hexane in extracting hydrophobic compounds such as carotenoid pigments, accumulated in persimmon fruits and peels from intermediate to late ripening stages [28].

These preliminary findings underscore the potential value of fruit peel, often discarded as agri-food waste, as a source of biologically active compounds. In particular, the high yield observed in mature samples suggests a significant content of polyphenolic compounds and carotenoids, positioning fruit peel as a promising resource for the nutraceutical industry.

### 3.2. Colorimetric Analysis

Colorimetric analysis revealed significant differences in CIEL*a*b* parameters, in relation, presumably, to the content of bioactive pigments such as carotenoids and flavonoids. The results for the homogenized and extracted samples are shown in Table 1.

The parameter L*, which measures brightness, was higher (about 20–30%) in immature homogenized samples than in mature and intermediate ripened samples, reflecting a lighter coloration, presumably associated with a lower concentration of visible pigments.

**Figure 2:**
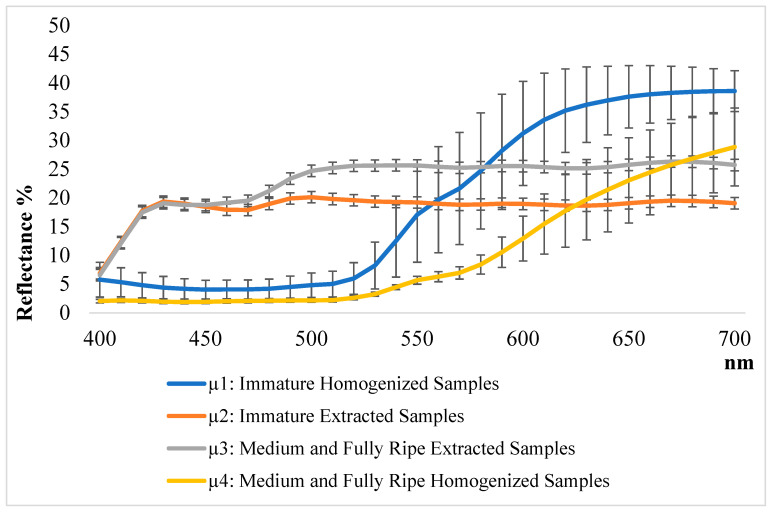
Reflectance curves representing the average spectral behavior of different persimmon samples at various ripening stages. The curves are calculated as the arithmetical average (µ) of µ1 (blue line—immature homogenized samples W_1_, F_1_, P_1_), µ2 (orange line—immature extracted samples WH_1_, FH_1_, PH_1_, WO_1_, FO_1_, PO_1_), µ3 (grey line—medium and fully ripe extracted samples WH_2,3_; FH_2,3_; PH_2,3_; WO_2,3_; FO_2,3_; PO_2-3_), and µ4 (yellow line—medium and full ripe homogenized sample W_2-3_; F_2-3_; P_2-3_).

These results align with those reported by Rodrigo et al. (2013) [30], who observed similar behavior in orange samples, in which the decrease in brightness correlated with the increase in carotenoids during ripening, intensifying the orange color. Differently, however, it appears in the immature samples of both hydroalcoholic and organic extracts, where the L* parameter decreases by 15–12% on average. This phenomenon is because hydroalcoholic and organic extracts, by concentrating polyphenols and carotenoids in solution (highly reflective pigments), tend to cause immature samples, containing fewer pigments than mature samples, to exhibit lower L* values than mature samples. These results are in line with other studies reporting a correlation between colorimetric parameters and pigments, confirming the utility of colorimetry as a useful nondestructive technique for estimating the content of bioactive compounds in fruits [19,22,31]. The different trends are also highlighted by the reflectance curves in Figure 2.

The calculated mathematical averages, related to the reflectance curves of the samples, were considered for exemplification: µ1-immature homogenized samples (media related: W_1_; F_1_; P_1_); µ2-immature extracted samples (media related: WH_1_; FH_1_; PH_1_; WO_1_; FO_1_; PO_1_); µ3-mature and intermediate ripening extracted samples (media related: WH_2-3_; FH_2-3_; PH_2-3_; WO_2-3_; FO_2-3_; PO_2-3_); µ4-mature and intermediate ripening homogenized samples (media related: W_2-3_; F_2-3_; P_2-3_).

The elevated standard deviations observed in the extracted samples reflect the heterogeneity in carotenoid content and the variability associated with the extraction process due to differences in sample composition and solubility of carotenoids. The reflectance curves related to homogenized samples (µ1 and µ4) exhibit a saturation curve pattern, reaching a maximum of absorption (around 600 nm) beyond which the transmittance remains constant. The curves related to the extracted samples (µ2 and µ3) exhibit a peak curve followed by a plateau, at 450–500 nm, corresponding to the maximum carotenoid absorption.

In addition, as shown by the values reported in Table 1, the high intensity of a* in ripe peels (P_3_, 34.11) suggests a high concentration of carotenoids, such as β-cryptoxanthin and lutein, responsible for the orange and red hues. This phenomenon has also been observed in other carotenoid-rich fruits, such as tomato, papaya, and goji berries, where orange-red pigments increase with ripening and are associated with high values of a* and b*, indicating, respectively, the red and yellow components [19,32].

The persimmon, particularly in its peel, follows a pattern like other fruits during ripening. As the fruit matures, the a* parameter (representing the red component) increases, indicating an accumulation of carotenoids. This makes the color analysis a useful method for monitoring pigment changes during fruit maturation. Additionally, the peel of ripe persimmons becomes browner, indicating a higher carotenoid content, confirmed by the increase in the a* parameter, but also a possible action of the polyphenol oxidase [19,33,34]. The b* parameter (representing the yellow component) is higher in unripe fruits, as shown by sample F_1_ (the highest value of b* of 42.35). This behavior is like that of other yellow-fleshed fruits, such as mango and papaya, where high b* values were associated with the presence of flavonoids and xanthophylls, responsible for this characteristic yellow-orange color.

**Figure 3:**
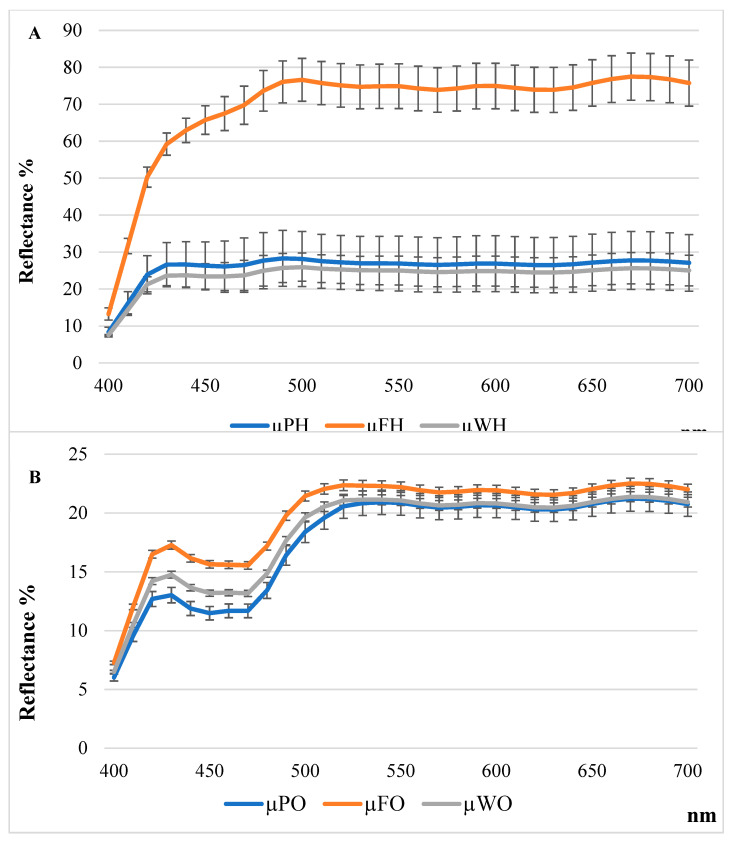
Reflectance curves related to hydroalcoholic extracts (**A**) and to organic extracts (**B**).

The situation changes after the extraction process. In this case, the a* parameter becomes negative in all samples, indicating a loss of red component and the appearance of a faint green nuance. This could be due to the co-extraction of chlorophyll pigments, interfering with the orange coloration of carotenoids [35]. For this reason, the parameter C*_ab_, the color saturation representing the whole pigment content, was better investigated [22]. Higher C*_ab_ values suggest a greater presence of pigments such as carotenoids and anthocyanins, contributing to more vivid and saturated colors [19,22,31]. Results deriving from color analysis (Figure 3) underline that, in the case of organic extracts, carotenoids are entirely produced during medium and full ripeness (WO_2_: 20.56 and WO_3_. 20.38 vs. WO_1_ 2.22; FO_2_. 15.40 and FO_3_ 17.34 vs. FO_1_ 1.40; PO_2_: 24.34 and PO_3_. 22.03 vs. PO_1_ 7.15); the same trend is shown in the polyphenols extracts by the peels, as well as the polyphenols content of the whole fruit and flesh suffers less evident variation during the three stages.

### 3.3. HPLC-DAD Analysis

**Figure 4:**
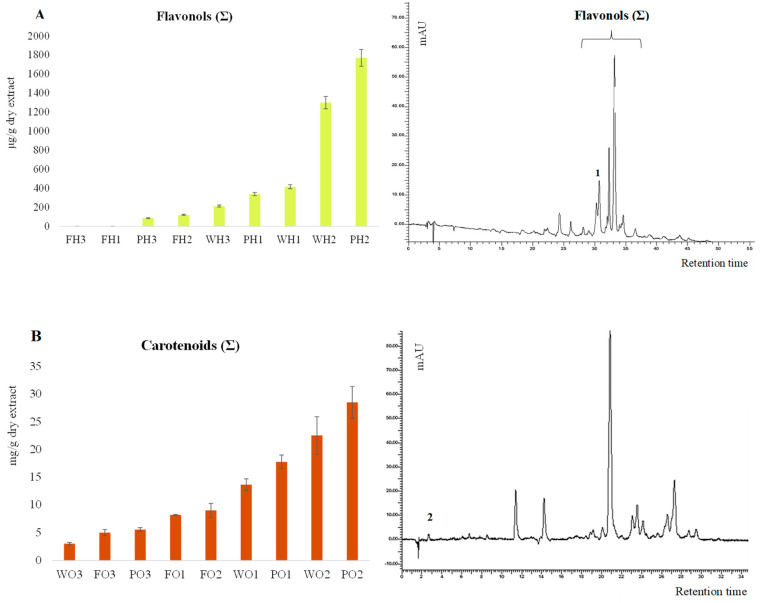
Examples of chromatograms monitored at 360 nm ((**A**): 1. rutin) and 450 nm ((**B**): 2. lutein).

All hydroalcoholic and organic extracts were subjected to HPLC-DAD analysis. The results are presented in Table 2. Chromatograms of the hydroalcoholic extracts were recorded at 360 nm, whereas the organic extracts were recorded at 450 nm (Figure 4).

For polyphenol analysis (Figure 4A), HPLC-DAD analysis revealed a non-uniform distribution of flavonols, quantified as a sum and expressed as µg/g rutin equivalents (identified using an external standard), across the different persimmon fractions based on ripening stage. As shown in Table 2, rutin concentration peaks in the peel samples (PH_3_ and PH_2_), with values of 118.7 µg/g and 176.5 µg/g for mature and intermediate peels, respectively, while in mature and intermediate pulp samples, rutin ranges from 118 µg/g to 250 µg/g. In the mature and intermediate whole fruit fraction (WH_3_ and WH_2_), total flavonols reach 416.5 µg/g and 1299.1 µg/g, representing a balance between peel and pulp contributions. These findings indicate that mature peel is the richest fraction in flavonols, whereas the pulp contains considerably lower amounts, suggesting a primary role of the peel in defense against oxidative and environmental stress [21,29,36]. The results obtained are in line with what has been reported in the literature [37,38].

Carotenoid content was quantified as the sum of peak areas, expressed as mg/g of dry extract (DE), using a lutein calibration curve. As shown in Table 2, lutein (Tr = 2.7) was identified in all the analyzed samples except PO_1_. This may be attributed to the fruit’s ripening stage, which allows the accumulation of certain carotenoids over others [39]. According to the literature [28,40], the carotenoids most commonly found in the fruit are zeaxanthin, β-cryptoxanthin, α-carotene, and β-carotene and could be part of the chromatographic profile shown in Figure 4B.

Lutein levels ranged from a minimum of 0.01 mg/g DE in FO_1_ to a maximum of 0.57 mg/g DE in PO_3_. The obtained data suggest that lutein concentration is elevated only in fully ripened samples, particularly in WO_2_, WO_3_, and PO_3_, representing 1–4% of total carotenoids. This trend is consistent when considering the total carotenoid content as well. The range varies from a minimum of 3 mg/g DE in WO_1_ to a maximum of 28 mg/g DE in PO_2_. As illustrated in Figure 4B, the samples of whole fruit and peels (PO_3_, WO_2_, and PO_2_) display the highest total carotenoid levels. These results align with the literature, indicating that carotenoid concentrations significantly increase in fruit peels during ripening, impacting both the color and nutritional content of mature fruit [41,42]. Different carotenoids have been associated with different health properties; in fact, apart from the most well-known provitamin A, carotenoids like β-carotene, α-carotene, β-cryptoxanthin, lutein, and zeaxanthin have been associated with the prevention of age-related macular degeneration [43,44]. These properties make the carotenoids ideal for the always-increasing functional food industry, as well as promoting the consumption of the natural products in which they are contained. Based on the findings presented, our results show total carotenoid yields consistent with those reported in other published studies by Bordiga et al. (2019) and Gea-Botella et al. (2021) [40,45].

**Figure 5:**
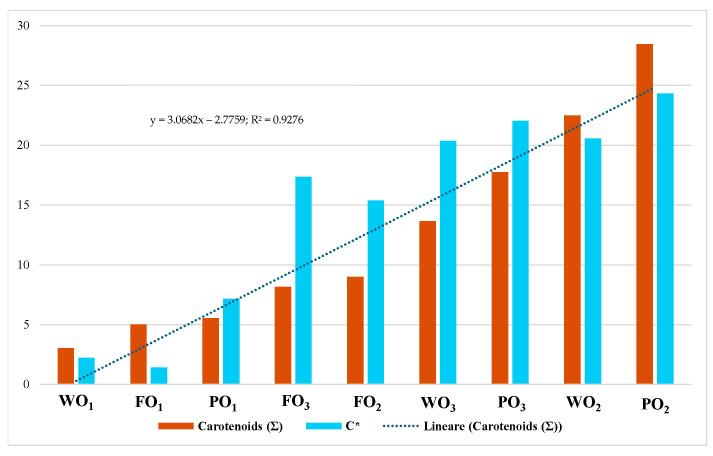
Linear regression between carotenoids data and C*_ab_ data.

The HPLC-DAD analysis of carotenoids in different *Diospyros kaki* samples reveals that their concentration varies depending on the ripeness stage and the considered fruit portion (skin, pulp, or whole fruit). Notably, the skin of ripe and intermediate fruits exhibits the highest levels of carotenoids, particularly β-cryptoxanthin and lutein, which contribute to the fruit’s characteristic orange-red hues. These findings are corroborated by colorimetric analysis, which shows a significant increase in the a* (red) and b* (yellow) values in these samples, indicating a higher density of carotenoid pigments. The chroma is high in the ripe skin samples, suggesting an increase in color saturation in parallel with the accumulation of carotenoids. Indeed, a good linear regression correlates the HPLC-DAD data and the C*_ab_ parameter (R^2^ = 0.9276) (Figure 5).

These results agree with other studies showing that colorimetric parameters (particularly a* and b*) increase with carotenoid accumulation during the ripening of fruits, such as kaki, tomato, and orange, and demonstrate that colorimetry can be a rapid and non-destructive method to estimate carotenoid content [19,30,32]. The highest chroma values in ripe peel samples suggest an increase in color saturation associated with carotenoid accumulation, confirming what was reported by Cairone et al. (2020) [22] in the context of quality control of fruits bearing high nutritional value. The observed correlation underlines the usefulness of colorimetry for indirectly estimating carotenoid content, allowing for the rapid identification of samples richer in bioactive compounds, favoring both nutraceutical applications and by-product valorization strategies. The correlation, between CIEL*a*b* parameters and carotenoid content, suggests that colorimetric analysis can serve as a rapid and non-destructive screening tool, as well as HPLC-DAD is essential for accurately determining carotenoid composition. Future studies should further validate the predictive accuracy of applied colorimetric methods by integrating them with additional chromatographic and spectroscopic techniques and results.

### 3.4. Antifungal Activity

The antimicrobial activity of *Diospyros kaki* extracts against *C. albicans*, as demonstrated in this study, reveals a selective behavior closely related to the fruit’s phytochemical composition, which varies depending on the maturation stage and the analyzed fruit part. Hydroalcoholic extracts FH_1_ (immature pulp) and WH_1_ (immature whole fruit) exhibited moderate antifungal activity, compared to fluconazole, which showed MIC values of 2 µg/mL. The extracts had MIC values of 250 µg/mL against both tested *C. albicans* strains, likely due to significant concentrations of flavonols such as rutin, detected in these samples (116–213 µg/g). Conversely, most other extracts showed negligible activity (MIC > 500 µg/mL). Additionally, enzymatic processes occurring during ripening may alter the chemical structure of bioactive compounds, reducing their antifungal potency despite higher total concentrations. These elements suggest that flavonoid activity in *Diospyros kaki* extracts depends not only on concentration but also on molecular complexity, synergistic interactions, and extractability under assay conditions [1,46,47,48]. On the contrary, carotenoids such as lutein and β-cryptoxanthin, prevalent in PO_2_ and WO_2_ extracts, did not enhance antifungal effects, suggesting that carotenoids may contribute more to stability and overall, to bioactivity than direct fungal inhibition. This aligns with previous literature indicating that polyphenolic compounds, including flavonols, play a key role in disrupting fungal cell walls and inhibiting microbial adhesion through oxidative stress induction and membrane disruption.

**Figure 6:**
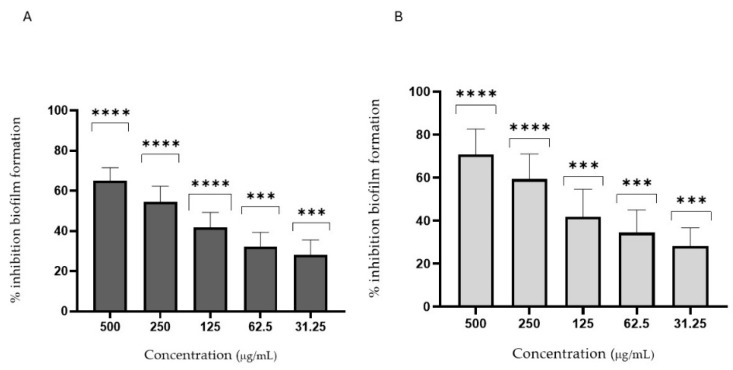
Activity of extracts against *Candida albicans* biofilm formation. Two experiments were performed in triplicate. Results expressed as mean ± standard deviation: (**A**) WH_1_; (**B**) FH_1_. *p* < 0.0001 very highly significant (****), 0.0001 ≤ *p* < 0.001 highly significant (***).

**Figure 7:**
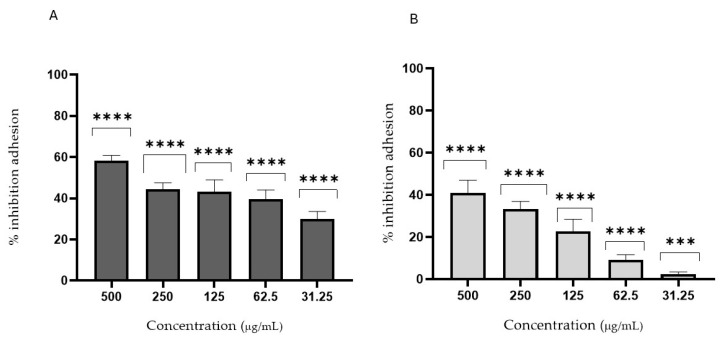
Activity of extracts against *Candida albicans* biofilm adhesion. Adhesion time: 90 min. Two experiments were performed in quadruplicate. Values expressed as mean percentage ± standard deviation. (**A**) WH_1_; (**B**) FH_1_. *p* < 0.0001 very highly significant (****), 0.0001 ≤ *p* < 0.001 highly significant (***).

Immature fruit extracts performed better than mature ones, likely due to the presence of bioactive secondary metabolites such as tannins and simpler polyphenols, not analyzed in this work, that are more bioavailable in early maturation stages. These compounds were previously studied for their ability to inhibit *C. albicans*, also inhibiting fungal enzymes and biofilm formation [49,50]. Hence, these hypotheses were evaluated by biofilm formation and adhesion tests (Figure 6 and Figure 7). FH_1_ and WH_1_ showed moderate inhibition of early *C. albicans* colonization. In fact, Figure 6 highlights the anti-biofilm efficacy of *Diospyros kaki* extracts, FH_1_ and WH_1_, maintaining inhibition rates exceeding 40–60% even at concentrations of 250 µg/mL. Moreover, to determine whether the inhibition of biofilm formation was due to the suppression of its initial stage—adhesion—the inhibition of cell adhesion was assessed. Figure 7 illustrates the anti-adhesion activity of FH_1_ and WH_1_, with inhibition rates between 45% and 65% at concentrations of 250–500 µg/mL. These properties indicate that key bioactive compounds effectively interfere with *C. albicans* initial adhesion, potentially preventing early biofilm formation and supporting their use in antifungal preventive strategies [51,52]. Although its activity is lower than that of fluconazole, it remains noteworthy since it derives from a food extract. The ability to control fungal growth could be particularly relevant in cases of dysbiosis. In particular, the extract’s effectiveness in inhibiting not only fungal growth but also adhesion and biofilm formation is especially promising for its potential use in preventing *Candida* growth and adhesion at the intestinal and skin levels.

**Figure 8:**
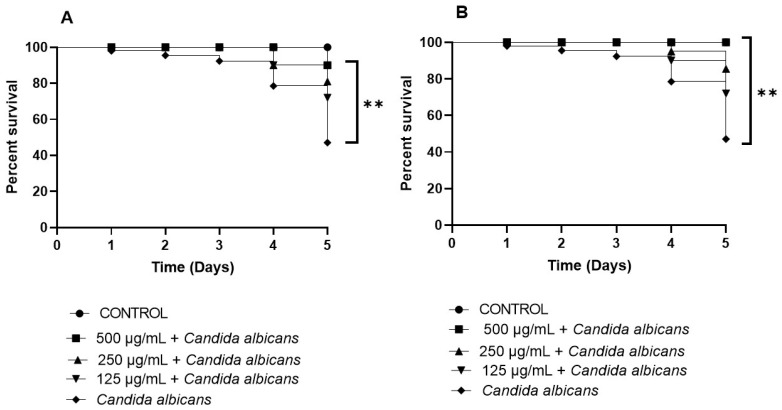
*G. mellonella* survival rate after *Candida albicans* infection and treatment with WH_1_ (**A**) and FH_1_ (**B**). *G. mellonella* survival was displayed via Kaplan–Meier curves with a curve comparison test; *p*-value: 0.0001 ≤ *p* < 0.001 highly significant (**).

The two selected active extracts against *C. albicans* were also tested in *Galleria mellonella* infected with *Candida* to assess their efficacy in an in vivo model. *G. mellonella* is widely used in preclinical research due to its structural and functional similarities with the mammalian immune system, including phagocytosis and superoxide production. Plasmatocytes and granulocytes in insects share functional roles with macrophages and dendritic cells in humans, while coagulation mechanisms also show parallels. Additionally, *G. mellonella* possesses a homologous receptor to mammalian C-reactive protein, enabling pathogen recognition. This model has been effective in identifying antimicrobial compounds, often providing results comparable to murine models. However, its main limitation lies in the absence of an adaptive immune response, as insects lack antibody production and rely solely on non-specific immunity [53]. Despite this, *G. mellonella* remains a valuable tool for preliminary in vivo studies on antifungal activity. Furthermore, Figure 8 shows the protective effect of FH_1_ and WH_1_ extracts in a *G. mellonella* survival assay. Larvae treated with these extracts exhibited significantly improved survival rates, exceeding 60% at the highest tested concentration of 500 µg/mL. This dose-dependent in vivo effect suggests that the antifungal activity of FH_1_ and WH_1_ extracts may result from a reduction in fungal virulence. The combined in vivo and in vitro findings support their potential application in the prevention of *Candida*-related infections. *C. albicans* is a commensal component of the microbiota in various anatomical sites, such as the intestine; however, uncontrolled proliferation and biofilm formation can lead to pathogenicity. Thus, targeting *Candida* proliferation and biofilm development may represent a promising strategy for infection control in these environments, while also potentially reducing the risk of resistance.

Moreover, the anti-adhesive properties of these extracts warrant further investigation for their potential use as protective barriers in food packaging, food preservation, and healthcare applications. The development of natural, eco-friendly antifungal agents is crucial for sustainable surface hygiene, offering a safer and more biocompatible alternative to synthetic fungicides.

These results underscore the significance of considering both the fruit part and the maturation stage to maximize the antimicrobial potential of *Diospyros kaki* extracts. Further research should target compound identification via LC-MS, broader pathogen screening, and extract combinations to assess synergistic effects, enhancing the use of *Diospyros kaki* as a sustainable source for nutraceutical and pharmaceutical applications while supporting agro-industrial by-product valorization.

## 4. Conclusions

The results of this preliminary study highlight the potential of *Diospyros kaki* extracts as sources of high-value bioactive compounds. Colorimetric and HPLC-DAD analyses revealed a correlation between carotenoid content and CIEL*a*b* parameters, suggesting that colorimetric properties may serve as useful indicators of bioactive compound levels.

Hydroalcoholic extracts derived from the immature pulp (FH_1_) and the immature whole fruit (WH_1_) demonstrated significant antifungal activity against *C. albicans*, by inhibiting biofilm formation and fungal adhesion. Chemical analyses confirmed a ripeness-dependent distribution of bioactive compounds, with the immature fruit showing higher antifungal efficacy despite a lower total polyphenol content than mature samples. This effect may be due to higher bioavailability or to simpler chemical structures of phenolic compounds in the immature samples, which allow better interactions with fungal cells.

The results we obtained in this study could represent the basis for toxicity studies and further studies that are needed for their possible use in the prevention of *Candida* colonization, reducing resistance problems. Refinement of extraction and purification methods could optimize the yield of compounds while respecting sustainability principles, aligning with circular economy strategies for agro-industrial waste reduction.

The protective effect observed in the *G. mellonella* survival test further supports the potential of these extracts for antifungal applications. The tested extracts interfere with the adhesion and maturation phases of biofilm formation, key characteristics of *Candida* biofilm for the resistance to antifungal drugs, and the ability to facilitate the spread of new infections.

## Figures and Tables

**Table 1:** Colorimetric data related to homogenized samples (W = whole, F = flesh, P = pulp), hydroalcoholic (WH, FH, PH), and organic extracts (WO, FO, PO).

Samples	Extraction Yield %	L*	a*	b*	C*_ab_	h_ab_
W_1_	- ^$^	51.62 ± 1.21 ^a^	26.46 ± 0.65 ^a^	45.83 ± 1.80 ^a^	52.92 ± 0.20 ^a^	59.99 ± 0.45 ^a^
W_2_	-	31.22 ± 0.30 ^b^	23.66 ± 6.38 ^a^	27.28 ± 4.39 ^b^	36.13 ± 7.50 ^b^	49.40 ± 3.15 ^a^
W_3_	-	30.94 ± 9.23 ^b^	24.90 ± 4.36 ^a^	24.41 ± 5.25 ^b^	35.20 ± 0.56 ^b^	44.35 ± 11.09 ^a^
F_1_	-	54.05 ± 0.52 ^a^	23.68 ± 1.17 ^a^	42.35 ± 1.79 ^a^	48.52 ± 0.90 ^a^	60.79 ± 1.48 ^a^
F_2_	-	28.74 ± 1.70 ^b^	18.21 ± 0.26 ^a^	23.61 ± 0.82 ^b^	29.82 ± 0.49 ^b^	52.35 ± 1.37 ^b^
F_3_	-	26.81 ± 4.88 ^b^	19.24 ± 4.15 ^a^	22.91 ± 4.60 ^b^	30.23 ± 0.84 ^b^	49.83 ± 11.67 ^b^
P_1_	-	37.95 ± 1.32 ^a^	31.66 ± 0.32 ^a^	35.14 ± 1.21 ^a^	47.30 ± 0.43 ^a^	47.98 ± 1.29 ^a^
P_2_	-	36.09 ± 2.21 ^a^	32.34 ± 0.74 ^a^	32.42 ± 3.39 ^a^	45.84 ± 1.88 ^a^	45.00 ± 3.65 ^a^
P_3_	-	33.31 ± 9.78 ^a^	34.11 ± 1.51 ^a^	33.46 ± 12.95 ^a^	48.13 ± 10.07 ^a^	43.40 ± 10.00 ^a^
WH_1_	4.68	52.43 ± 0.25 ^a^	−1.77 ± 1.38 ^a^	1.35 ± 0.56 ^a^	2.23 ± 1.09 ^a^	142.61 ± 0.22 ^a^
WH_2_	6.47	59.86 ± 2.87 ^a^	−1.88 ± 0.45 ^a^	2.49 ± 0.97 ^a^	3.12 ± 1.04 ^a^	127.85 ± 4.37 ^b^
WH_3_	5.09	58.17 ± 0.91 ^a^	−2.23 ± 0.21 ^a^	4.22 ± 1.75 ^b^	4.79 ± 1.63 ^a^	119.24 ± 8.08 ^b^
FH_1_	3.54	50.31 ± 1.05 ^a^	−1.07 ± 0.62 ^a^	−0.82 ± 1.49 ^b^	1.35 ± 0.66 ^b^	217.66 ± 1.45 ^a^
FH_2_	10.02	60.97 ± 1.37 ^b^	−1.91 ± 0.52 ^a^	3.30 ± 1.52 ^a^	3.82 ± 1.58 ^a^	121.14 ± 5.24 ^b^
FH_3_	11.03	64.04 ± 2.86 ^b^	−1.47 ± 0.14 ^a^	1.27 ± 1.93 ^b^	2.27 ± 0.98 ^a^	150.51 ± 46.75 ^b^
PH_1_	5.58	50.80 ± 1.33 ^a^	−1.52 ± 0.07 ^a^	0.78 ± 1.09 ^a^	1.71 ± 0.89 ^a^	152.78 ± 1.11 ^a^
PH_2_	8.34	56.46 ± 0.07 ^b^	−3.38 ± 0.02 ^b^	8.40 ± 2.28 ^b^	9.07 ± 2.12 ^b^	112.54 ± 5.44 ^a^
PH_3_	13.83	62.36 ± 2.50 ^b^	−3.55 ± 0.28 ^b^	7.69 ± 1.63 ^b^	8.47 ± 1.60 ^b^	115.08 ± 2.99 ^a^
WO_1_	0.02	49.52 ± 1.45 ^a^	−1.62 ± 0.23 ^a^	1.52 ± 1.35 ^a^	2.22 ± 0.14 ^a^	136.81 ± 1.63 ^a^
WO_2_	0.09	51.64 ± 2.12 ^a^	−4.43 ± 0.57 ^b^	20.07 ± 1.81 ^b^	20.56 ± 1.89 ^b^	102.42 ± 0.45 ^a^
WO_3_	0.04	54.68 ± 1.40 ^a^	−4.97 ± 0.20 ^b^	19.77 ± 1.11 ^b^	20.38 ± 0.73 ^b^	104.11 ± 1.14 ^a^
FO_1_	0.01	50.66 ± 0.86 ^a^	−1.39 ± 1.43 ^a^	0.17 ± 1.16 ^a^	1.40 ± 0.44 ^a^	172.92 ± 1.93 ^a^
FO_2_	0.04	53.01 ± 1.03 ^a^	−4.00 ± 0.74 ^b^	14.86 ± 4.82 ^b^	15.40 ± 4.84 ^b^	105.41 ± 2.11 ^b^
FO_3_	0.04	56.50 ± 0.26 ^a^	−4.58 ± 1.88 ^b^	16.72 ± 1.75 ^b^	17.34 ± 0.38 ^b^	105.31 ± 1.31 ^b^
PO_1_	0.02	51.27 ± 1.15 ^a^	−2.67 ± 1.82 ^a^	6.64 ± 0.56 ^a^	7.15 ± 1.22 ^a^	111.92 ± 1.38 ^a^
PO_2_	0.09	48.70 ± 1.56 ^a^	−3.91 ± 0.22 ^a^	24.02 ± 2.02 ^b^	24.34 ± 2.03 ^b^	99.25 ± 0.25 ^a^
PO_3_	0.08	54.35 ± 1.26 ^a^	−5.00 ± 0.66 ^b^	21.45 ± 1.34 ^b^	22.03 ± 1.38 ^b^	103.13 ± 0.30 ^a^

**_1,2,3_** Correspond to immature, medium mature, and ripe fruits. ^a,b^ Values with the same letter within each group (W, P, F) indicate no statistically significant differences among 1, 2, and 3 (*p* value < 0.05). ^$^ The sample was subjected to colorimetric analysis as such, without any extraction or adulteration of the sample. The obtained data of the extraction yield are the result of three experimental measurements (within ± 3%).

**Table 2:** HPLC-DAD data of hydroalcoholic (**H**) and organic extracts (**O**).

µg/g Dry Extract	Rutin	Flavonols (Σ)	mg/g Dry Extract	Lutein	Carotenoids (Σ)
**PH_1_**	86.4 ± 2.6 ^a^	86.4 ± 4.3 ^a^	**PO_1_**	BDL *	5.54 ± 0.36 ^a^
**PH_2_**	176.5 ± 5.3 ^b^	1767.9 ± 88.3 ^b^	**PO_2_**	0.20 ± 0.02 ^a^	28.47 ± 2.85 ^b^
**PH_3_**	118.7 ± 3.6 ^b^	338.7 ± 16.9 ^b^	**PO_3_**	0.57 ± 0.03 ^a^	17.74 ± 1.24 ^b^
**FH_1_**	116.1 ± 0.1 ^a^	116.1 ± 0.1 ^a^	**FO_1_**	0.01 ± 0.002 ^a^	5.00 ± 0.56 ^a^
**FH_2_**	121.5 ± 3.6 ^a^	121.5 ± 6.1 ^a^	**FO_2_**	0.09 ± 0.01 ^a^	9.00 ± 1.26 ^a^
**FH_3_**	118.2 ± 0.1 ^a^	118.2 ± 0.1 ^a^	**FO_3_**	0.11 ± 0.01 ^a^	8.18 ± 0.08 ^b^
**WH_1_**	213.6 ± 6.4 ^a^	213.6 ± 10.7 ^a^	**WO_1_**	0.11 ± 0.01 ^a^	3.03 ± 0.20 ^a^
**WH_2_**	247.4 ± 7.4 ^a^	1299.1 ± 65.0 ^b^	**WO_2_**	0.30 ± 0.05 ^b^	22.49 ± 3.37 ^b^
**WH_3_**	155.4 ± 4.7 ^b^	416.5 ± 20.8 ^b^	**WO_3_**	0.39 ± 0.02 ^b^	13.64 ± 1.05 ^b^

^a,b^ Values with the same letter within each group (P, F, W) indicate no statistically significant differences among 1, 2, and 3 (*p* value < 0.05); * Below Detection Limit.

## Data Availability

The original contributions presented in the study are included in the article, further inquiries can be directed to the corresponding author.
